# Supplementary material for: MacroH2A restricts inflammatory gene expression in melanoma cancer-associated fibroblasts by coordinating chromatin looping
Source: Nat Cell Biol. 2023 Aug 21;25(9):1332–45. doi: 10.1038/s41556-023-01208-7 (PMC10495263; doi:10.1038/s41556-023-01208-7)
Supplement: Supplementary file 1 — Reporting Summary [file 41556_2023_1208_MOESM1_ESM.pdf]

nature portfolio

Corresponding author(s): Emily Bernstein, Dan Filipescu

Last updated by author(s): Jun 12, 2023

Reporting Summary

Nature Portfolio wishes to improve the reproducibility of the work that we publish. This form provides structure for consistency and transparency in reporting. For further information on Nature Portfolio policies, see our Editorial Policies and the Editorial Policy Checklist.

Statistics

For all statistical analyses, confirm that the following items are present in the figure legend, table legend, main text, or Methods section.

n/a	Confirmed	
☐	☒	The exact sample size (*n*) for each experimental group/condition, given as a discrete number and unit of measurement
☐	☒	A statement on whether measurements were taken from distinct samples or whether the same sample was measured repeatedly
☐	☒	The statistical test(s) used AND whether they are one- or two-sided<br>*Only common tests should be described solely by name; describe more complex techniques in the Methods section.*
☒	☐	A description of all covariates tested
☐	☒	A description of any assumptions or corrections, such as tests of normality and adjustment for multiple comparisons
☐	☒	A full description of the statistical parameters including central tendency (e.g. means) or other basic estimates (e.g. regression coefficient) AND variation (e.g. standard deviation) or associated estimates of uncertainty (e.g. confidence intervals)
☐	☒	For null hypothesis testing, the test statistic (e.g. *F*, *t*, *r*) with confidence intervals, effect sizes, degrees of freedom and *P* value noted<br>*Give P values as exact values whenever suitable.*
☒	☐	For Bayesian analysis, information on the choice of priors and Markov chain Monte Carlo settings
☒	☐	For hierarchical and complex designs, identification of the appropriate level for tests and full reporting of outcomes
☐	☒	Estimates of effect sizes (e.g. Cohen's *d*, Pearson's *r*), indicating how they were calculated

Our web collection on statistics for biologists contains articles on many of the points above.

Software and code

Policy information about availability of computer code

Data collection	Flow cytometry: FacsDiVa (BD Biosciences) v8.0.2<br>qPCR: CFX Manager (Bio-Rad) v3.1<br>Automated microscopy: Gen5 (Agilent) v3.12
Data analysis	No custom code was generated for this study. All scripts used for bulk RNA seq, scRNA seq, ATAC seq, ChIP seq and CUT&RUN data analysis in this study are available from the corresponding author upon request.<br><br>Flow cytometry:<br>FCS Express (De Novo Software) v7.12<br><br>Statistics:<br>Prism (GraphPad) v9.5.1<br><br>RNA-seq:<br>salmon v1.2.1<br>DESeq2 v1.36.0<br>fgsea v1.22.0<br>HOMER v4.10<br>limma v3.54.0<br><br>scRNA_seq:

cellranger v7.0.1 (10X Genomics)
Seurat v4.0
SCPA v1.5.1
Monocle3 v1.3.1
singleR v1.10.0
miloR v1.5.0
Augur v1.0.3
CellChat v1.6.1
SeuratWrappers v0.3.0
SingleCellExperiment v1.12
PerformanceAnalytics v2.0.4

Spatial transcriptomics:
spaceranger v2.0.0 (10X Genomics)
psych v2.3.3

TCGA data analysis:
TCGAbiolinks v2.25.3
edgeR v3.40.1
IOBR v0.99.9

ChIP-seq, CUT&RUN, ATAC-seq:
NGmerge v.0.3
bowtie2 v.2.4.1
samtools v.1.9
deepTools v.3.5.1
MACS2 v.2.1.0
bedtools v.2.29.2
Trimmomatic v.0.36
ROSE v0.11
SEACR v.1.3

Micro-C:
BWA v0.7.17-r1188
PairTools v1.0.2
CHiCAGO v1.2.0
UCSC-Utils v2.9

For manuscripts utilizing custom algorithms or software that are central to the research but not yet described in published literature, software must be made available to editors and reviewers. We strongly encourage code deposition in a community repository (e.g. GitHub). See the Nature Portfolio guidelines for submitting code & software for further information.

Data

Policy information about availability of data

All manuscripts must include a data availability statement. This statement should provide the following information, where applicable:

- Accession codes, unique identifiers, or web links for publicly available datasets
- A description of any restrictions on data availability
- For clinical datasets or third party data, please ensure that the statement adheres to our policy

The transcriptomic and epigenomic datasets including raw and processed sequencing data generated and analyzed during the current study are available in the GEO repository under accession number GSE200751 (https://www.ncbi.nlm.nih.gov/geo/query/acc.cgi?acc=GSE200751) and in this article's table files. TCGA melanoma data is publicly available through the NCI Genomic Data Commons (GDC) data portal under project ID TCGA-SKCM (https://portal.gdc.cancer.gov/projects/TCGA-SKCM). The human pan-cancer scRNA-seq dataset mined in this study is available in the GEO repository under accession number GSE210347 (https://www.ncbi.nlm.nih.gov/geo/query/acc.cgi?acc=GSE210347). The mouse M25 (GRCm38.p6) genome assembly and gene set used for transcriptomic and epigenomic analysis are available at Gencode (https://www.gencodegenes.org/mouse/release_M25.html). All other data supporting the findings of this study is available from the corresponding author upon request.

Research involving human participants, their data, or biological material

Policy information about studies with human participants or human data. See also policy information about sex, gender (identity/presentation), and sexual orientation and race, ethnicity and racism.

Reporting on sex and gender	This study does not involve human participants or generate human data.
Reporting on race, ethnicity, or other socially relevant groupings	This study does not involve human participants or generate human data.
Population characteristics	This study does not involve human participants or generate human data.
Recruitment	This study does not involve human participants or generate human data.

Ethics oversight	This study does not involve human participants or generate human data.

Note that full information on the approval of the study protocol must also be provided in the manuscript.

Field-specific reporting

Please select the one below that is the best fit for your research. If you are not sure, read the appropriate sections before making your selection.

☒ Life sciences ☐ Behavioural & social sciences ☐ Ecological, evolutionary & environmental sciences

For a reference copy of the document with all sections, see nature.com/documents/nr-reporting-summary-flat.pdf

Life sciences study design

All studies must disclose on these points even when the disclosure is negative.

Sample size	Based on the nature of these studies, no sample-size calculation was performed. Sample size was chosen based on available number of animals or cell lines, attempting to maximize the number of biological replicates analyzed whenever possible.
Data exclusions	No data was excluded. Biological samples were excluded only as a result of sample preparation or data acquisition failure.
Replication	All experiments were replicated with the exception of Supplemental Figure 4n and o, H3K27ac ChIP-seq and macroH2A1 CUT&RUN. Multiple cohorts of mice were used as available due to limited breeding output and comparable mouse age required at induction in WT and dKO. Importantly, for tumor analysis experiments, each data point represents a distinct animal and therefore a biological replicate. For tumor immunophenotyping experiments, data shown is a sum of at least 2 experiments, each with at least 4 animals per genotype.<br>Tumor induction – Summary of 8 experiments, WT/dKO mice in each as follows: 7/6, 4/6, 4/5, 5/5, 4/4, 4/4, 5/5, 5/4.<br>RNA-seq – Summary of 3 experiments, 6 mice/genotype (2 in each experiment)<br>Sorted CD8 RNA-seq – 1 experiment, 4 mice/genotype<br>scRNA-seq – 3 experiments, 1/genotype in each experiment<br>Sorted CAFs RNA-seq – 1 experiment, 4 mice/genotype<br>Spatial transcriptomics – 1 experiment, 1 mouse/genotype<br>Human melanoma CAF macroH2A and cytokine quantification – macroH2A western blot 3 technical replicates, ELISA 8 technical replicates for 11 independent biological samples.<br>Sorted CAF ATAC-seq – 1 experiment, 2 mice/genotype<br>Cultured CAF Micro-C – 1 experiment, 2 technical replicates/condition<br>Supplementary Figure 4n was a confirmation at the protein level of extensively replicated transcriptional changes (Figures 4d, f), so we did not consider replicates were necessary.<br>The findings in figure 4o were confirmed by RNA-seq on replicates independent from this material in an experiment currently in Supplemental Figure 8b.<br>In our experience, and standard in the field, the highly reproducible nature of ChIP-seq does not require technical replicates. No additional CAF lines were available as biological replicates. MacroH2A1 cut&run was benchmarked against existing ChIP-seq data (Supplemental Figure 6a) and was highly similar.
Randomization	No samples were allocated to experimental groups as no treatments were tested on any type of subject in this study. Comparisons were performed on murine-derived samples according to the genotype of the animal they were derived from. Experiments not involving mice were based on molecular/cellular biology readouts where acquisition and analysis were performed automatically, in the same manner for all samples, and did not require randomization. For TCGA analysis, samples were allocated to primary/metastatic cohorts based on the appropriate recorded metadata, and into macroH2A high and low categories based on the 1st and 3rd tercile of MACROH2A1 or MACROH2A2 expression; no covariates were included in the analysis. For analysis of human melanoma CAFs, the available cell lines were grouped into MACROH2A2 high and low based on MACROH2A2 protein level measured by western blotting; no covariates were included in the analysis.
Blinding	For most experiments, blinding was not possible because the same investigator(s) who generated the mice determined their genotype, performed tumor induction, processed samples, acquired and analyzed the data. Histopathologic scoring of murine tumors was performed in a blinded manner, where information on mouse genotype, gender or age was withheld from pathologists. Experiments involving molecular/cellular biology readouts were acquired and analyzed automatically, using identical parameters for all samples, and therefore blinding was not relevant. Knowledge of sample genotype was necessary in order to perform the analysis.

Reporting for specific materials, systems and methods

We require information from authors about some types of materials, experimental systems and methods used in many studies. Here, indicate whether each material, system or method listed is relevant to your study. If you are not sure if a list item applies to your research, read the appropriate section before selecting a response.

Materials & experimental systems

n/a	Involved in the study	
☐	☒	Antibodies
☐	☒	Eukaryotic cell lines
☒	☐	Palaeontology and archaeology
☐	☒	Animals and other organisms
☒	☐	Clinical data
☒	☐	Dual use research of concern
☒	☐	Plants

Methods

n/a	Involved in the study	
☐	☒	ChIP-seq
☐	☒	Flow cytometry
☒	☐	MRI-based neuroimaging

Antibodies

Antibodies used	Antibody catalogue numbers, manufacturer websites and their dilutions are provided in Table 8 for all commercial antibodies, and a publication reference for one non-commercial antibody.<br>In addition to Table 8, the following antibodies were used for epigenomic profiling by ChIP and CUT&RUN, respectively:<br>H3K27ac: 13-0045, Epicypher, lot # 20120001-28, 4 µg/reaction<br>macroH2A1: ab37264, Abcam, lot # GR278020-1, 1 µg/reaction
Validation	Antibodies were validated by manufacturer, by publications cited by manufacturer, and in the case of the one non-commercial antibody, as detailed in our study for its relevant application by using macroH2A-deficient tissue. Links and species/application-relevant validation statements are provided in Table 8. In addition to Table 8:<br>Epicypher H3K27ac 13-0045 validation data is provided by manufacturer here https://www.epicypher.com/products/antibodies/snap-chip-certified-antibodies/histone-h3k27ac-antibody-snap-chip-certified ; antibody is validated for ChIP-Seq in mouse, among others.<br>Abcam macroH2A1 ab37264 validation data is provided for CUT&RUN in Supplemental Figure 6a.

Eukaryotic cell lines

Policy information about cell lines and Sex and Gender in Research

Cell line source(s)	Murine cells used in this study were derived directly from our mouse colony. Human melanoma CAF primary cultures were obtained from the NCI patient-derived models repository, and the laboratory of Dr. Andrew Aplin. Male and female CAFs (mouse and human) were used in this study.
Authentication	For murine CAFs, genotyping was performed for engineered genes of interest. Human CAFs were identified by positive detection of CAF-specific markers and non-detectable levels of melanoma markers.
Mycoplasma contamination	All cell lines were routinely tested for mycoplasma by PCR and were negative.
Commonly misidentified lines<br>(See ICLAC register)	No such cell lines were used.

Animals and other research organisms

Policy information about studies involving animals; ARRIVE guidelines recommended for reporting animal research, and Sex and Gender in Research

Laboratory animals	The strain used was generated in-house by breeding B6;FVB-Tg(Tyr-cre/ERT2)13Bos Braf<tm1Mmcm> Pten<tm1Rdp> and 129S6.Cg-Macroh2a2<tm1.1Peh> Macroh2a1<tm1Peh> or 129S6/SvEvTac mice. Mice were housed in a facility with specified pathogen-free (SPF) health status, in individually ventilated cages at 21-22ºC and 39-50% relative humidity, on a 7AM-7PM light cycle, with free access to food and water.
Wild animals	No wild animals are used in this study.
Reporting on sex	Animals of both sexes were included in the (immuno)phenotypic characterization of murine melanomas. Differences in tumor growth between sexes were tested for, and were found to be non-significant (Supplemental Figure 1b). Females were used for scRNA-seq and spatial transcriptomics, to avoid the potential impact of sex-specific transcripts on integration.
Field-collected samples	No field-collected samples are used in this study.
Ethics oversight	The Icahn School of Medicine at Mount Sinai's Institutional Animal Care and Use Committee approved the study protocol (Protocol # LA11-00122).

Note that full information on the approval of the study protocol must also be provided in the manuscript.

Plants

Seed stocks: No plants are used in this study

Novel plant genotypes: No plants are used in this study

Authentication: No plants are used in this study

ChIP-seq

Data deposition

☒ Confirm that both raw and final processed data have been deposited in a public database such as GEO.

☒ Confirm that you have deposited or provided access to graph files (e.g. BED files) for the called peaks.

Data access links
May remain private before publication.

https://www.ncbi.nlm.nih.gov/geo/query/acc.cgi?acc=GSE200751

Files in database submission

Accession Title Release date Status Supplementary files

GSE200751 MacroH2A restricts melanoma progression via inhibition of chemokine expression in cancer-associated fibroblasts. Aug 01, 2023 approved None
----
GSE200725 MacroH2A restricts melanoma progression via inhibition of chemokine expression in cancer-associated fibroblasts [RNA-seq] Aug 01, 2023 approved None
GSM6042770 Mouse # 64727 melanoma RNA-seq Aug 01, 2023 approved SF
GSM6042771 Mouse # 64747 melanoma RNA-seq Aug 01, 2023 approved SF
GSM6042772 Mouse # 64799 melanoma RNA-seq Aug 01, 2023 approved SF
GSM6042773 Mouse # 64803 melanoma RNA-seq Aug 01, 2023 approved SF
GSM6042774 Mouse # 64805 melanoma RNA-seq Aug 01, 2023 approved SF
GSM6042775 Mouse # 64808 melanoma RNA-seq Aug 01, 2023 approved SF
GSM6042776 Mouse # 64660 melanoma RNA-seq Aug 01, 2023 approved SF
GSM6042777 Mouse # 64661 melanoma RNA-seq Aug 01, 2023 approved SF
GSM6042778 Mouse # 64982 melanoma RNA-seq Aug 01, 2023 approved SF
GSM6042779 Mouse # 65018 melanoma RNA-seq Aug 01, 2023 approved SF
GSM6042780 Mouse # 65020 melanoma RNA-seq Aug 01, 2023 approved SF
GSM6042781 Mouse # 65041 melanoma RNA-seq Aug 01, 2023 approved SF
GSM6042782 Mouse # 65662 tumor-infiltrating CD8 RNA-seq Aug 01, 2023 approved SF
GSM6042783 Mouse # 65664 tumor-infiltrating CD8 RNA-seq Aug 01, 2023 approved SF
GSM6042784 Mouse # 65666 tumor-infiltrating CD8 RNA-seq Aug 01, 2023 approved SF
GSM6042785 Mouse # 65668 tumor-infiltrating CD8 RNA-seq Aug 01, 2023 approved SF
GSM6042786 Mouse # 65674 tumor-infiltrating CD8 RNA-seq Aug 01, 2023 approved SF
GSM6042787 Mouse # 65675 tumor-infiltrating CD8 RNA-seq Aug 01, 2023 approved SF
GSM6042788 Mouse # 65677 tumor-infiltrating CD8 RNA-seq Aug 01, 2023 approved SF
GSM6042789 Mouse # 65678 tumor-infiltrating CD8 RNA-seq Aug 01, 2023 approved SF
GSM6042790 Mouse # 66561 CAF RNA-seq Aug 01, 2023 approved SF
GSM6042791 Mouse # 66562 CAF RNA-seq Aug 01, 2023 approved SF
GSM6042792 Mouse # 66565 CAF RNA-seq Aug 01, 2023 approved SF
GSM6042793 Mouse # 66566 CAF RNA-seq Aug 01, 2023 approved SF
GSM6042794 Mouse # 66554 CAF RNA-seq Aug 01, 2023 approved SF
GSM6042795 Mouse # 66555 CAF RNA-seq Aug 01, 2023 approved SF
GSM6042796 Mouse # 66558 CAF RNA-seq Aug 01, 2023 approved SF
GSM6042797 Mouse # 66559 CAF RNA-seq Aug 01, 2023 approved SF
GSM6042798 iDF WT serum 0 min replicate 1 RNA-seq Aug 01, 2023 approved SF
GSM6042799 iDF WT serum 0 min replicate 2 RNA-seq Aug 01, 2023 approved SF
GSM6042800 iDF WT serum 30 min replicate 1 RNA-seq Aug 01, 2023 approved SF
GSM6042801 iDF WT serum 30 min replicate 2 RNA-seq Aug 01, 2023 approved SF
GSM6042802 iDF dKO serum 0 min replicate 1 RNA-seq Aug 01, 2023 approved SF
GSM6042803 iDF dKO serum 0 min replicate 2 RNA-seq Aug 01, 2023 approved SF
GSM6042804 iDF dKO serum 30 min replicate 1 RNA-seq Aug 01, 2023 approved SF
GSM6042805 iDF dKO serum 30 min replicate 2 RNA-seq Aug 01, 2023 approved SF

GSE200734 MacroH2A restricts melanoma progression via inhibition of chemokine expression in cancer-associated fibroblasts [epigenomics] Aug 01, 2023 approved None
GSM6042890 Mouse # 66620 CAF ATAC-seq Aug 01, 2023 approved BW
GSM6042891 Mouse # 66626 CAF ATAC-seq Aug 01, 2023 approved BW
GSM6042892 Mouse # 66623 CAF ATAC-seq Aug 01, 2023 approved BW
GSM6042893 Mouse # 66624 CAF ATAC-seq Aug 01, 2023 approved BW
GSM6042894 CAF culture from mouse # 66391 H3K27ac ChIP-seq Aug 01, 2023 approved BW
GSM6042895 CAF culture from mouse # 66383 H3K27ac ChIP-seq Aug 01, 2023 approved BW
GSM6042896 CAF culture genomic DNA input for ChIP-seq Aug 01, 2023 approved BW

GSM6042897 CAF culture from mouse # 66391 macroH2A1 CUT&RUN prior to serum stimulation Aug 01, 2023 approved BW
GSM6042898 CAF culture from mouse # 66391 macroH2A1 CUT&RUN 30 minutes after serum stimulation Aug 01, 2023 approved BW
GSM6042899 CAF culture from mouse # 66391 IgG background for CUT&RUN Aug 01, 2023 approved BW
GSM6042900 H3K27ac ChIP-seq in iDF dKO cells Aug 01, 2023 approved BW
GSM6042901 H3K27ac ChIP-seq in iDF WT cells Aug 01, 2023 approved BW
GSM6042902 iDF culture genomic DNA input for ChIP-seq Aug 01, 2023 approved BW

GSE229530 MacroH2A restricts melanoma progression via inhibition of chemokine expression in cancer-associated fibroblasts [scRNA-seq] Aug 01, 2023 approved RDATA RDATA
GSM7164976 Mouse #66193 scRNA-seq Aug 01, 2023 approved TSV TSV MTX
GSM7164977 Mouse #66208 scRNA-seq Aug 01, 2023 approved TSV TSV MTX
GSM7164978 Mouse #66691 scRNA-seq Aug 01, 2023 approved TSV TSV MTX
GSM7164979 Mouse #66675 scRNA-seq Aug 01, 2023 approved TSV TSV MTX
GSM7164980 Mouse #19332 scRNA-seq Aug 01, 2023 approved TSV TSV MTX
GSM7164981 Mouse #19323 scRNA-seq Aug 01, 2023 approved TSV TSV MTX

GSE229531 MacroH2A restricts melanoma progression via inhibition of chemokine expression in cancer-associated fibroblasts [3D chromatin] Aug 01, 2023 approved BIGINTERACT BIGINTERACT
GSM7164982 CAF culture from mouse # 66391 pcMicro-C replicate 1 Aug 01, 2023 approved None
GSM7164983 CAF culture from mouse # 66391 pcMicro-C replicate 2 Aug 01, 2023 approved None
GSM7164984 CAF culture from mouse # 66383 pcMicro-C replicate 1 Aug 01, 2023 approved None
GSM7164985 CAF culture from mouse # 66383 pcMicro-C replicate 2 Aug 01, 2023 approved None
----
GSE229532 MacroH2A restricts melanoma progression via inhibition of chemokine expression in cancer-associated fibroblasts [spatial transcriptomics] Aug 01, 2023 approved RDATA RDATA
GSM7164986 Mouse #19333 spatial transcriptomics Aug 01, 2023 approved H5 JSON PNG CSV
GSM7164987 Mouse #19331 spatial transcriptomics Aug 01, 2023 approved H5 JSON PNG CSV

Genome browser session
(e.g. UCSC)

https://genome.ucsc.edu/s/filipd02/CAF%20Filipescu%20v2
https://genome.ucsc.edu/s/filipd02/iDF%20Filipescu

Methodology

Replicates

In our experience, and standard in the field, the highly reproducible nature of ChIP-seq does not require technical replicates. No additional CAF lines were available as biological replicates. MacroH2A1 cut&run was benchmarked against existing ChIP-seq data (Supplemental Figure 6a) and was highly similar.

Sequencing depth

CAF H3K27ac ChIP - single end
Sample 66391_H3K27ac 66383_H3K27ac Inputs_combined
Total reads 39383904 51206219 70931993
Aligned to mouse 37083059 43981970 69796720
Filtered 30290128 39456067 46240441
Deduplicated 13464611 10736503 34167706

CAF macroH2A1 CUT&RUN - paired end
Sample 66591-U-m1 66591-S-m1 66391-IgG
Total reads 29912046 35920306 35371080
Aligned to mouse 26191146 31614828 26824800
Filtered 19569422 24532310 19528262
Deduplicated 19079270 22770678 17738696

iDF H3K27ac ChIP - single end
Sample iDF WT H3K27ac iDF dKO H3K27ac input mixed
Total reads 53295577 54864962 86801179
Aligned to mouse 36864021 39644575 86190317
Filtered 30730014 34341625 63956202
Deduplicated 24792362 26175630 61629179

Antibodies

H3K27ac: 13-0045, Epicypher, lot # 20120001-28, 4 µg/reaction

Peak calling parameters

```
#Read mapping:
bowtie2 -p ${PPN} -x $INDEXDIR --local --very-sensitive-local --no-unal -U ${sample}_out.fastq.gz 2> ${OUTDIR}${sample}_bowtie2.log -S ${sample}.sam --un ${sample}_unaligned.fastq
samtools sort -@ ${PPN}-1 ${sample}.sam -O bam -o ${sample}_sorted.bam
samtools index -@ ${PPN} ${sample}_sorted.bam
samtools view -@ ${PPN}-1 ${sample}_sorted.bam chr{1..19} chrX chrY -q 30 -b -o ${sample}_filtered.bam
samtools index -@ ${PPN} ${sample}_filtered.bam
samtools markdup -@ ${PPN}-1 -r -S ${sample}_filtered.bam ${sample}_dedup.bam
samtools index -@ ${PPN} ${sample}_dedup.bam

#Peak calling CAF:
macs2 callpeak -t H3K27ac-merge.bam -c Input.bam --name H3K27ac-1e-7 --extsize 300 --to-large --nomodel -f BAM -g 3.0e9 -s 75 -p
```

```
1e-7 --outdir /output/ --verbose 2

#Peak calling iDF:
macs2 callpeak -t iDF-master-WT_dKO-H3K27ac_dedup.bam -f BAM -c  iDF-mixed-input_dedup.bam --outdir /output/ -n iDF-master-WT_dKO-H3K27ac_q1e-9-g mm --extsize 300 -q 1e-9
```

Data quality

After removal of low-quality and duplicated reads, and peak calling on master bam files, ChIP-seq and input reads were normalized for sequencing depth using counts per million (CPM). CPM fold enrichment (FE) was calculated for peaks in each sample over input.

Peaks Above 5 FE Below 5 FE % above 5 fe
CAF WT 33700 6832 83.14%
CAF dKO 39162 1371 96.62%
IDF WT 30857 2386 92.82%
IDF DKO 30096 3146 90.54%

Software

Adapters were trimmed with Trimmomatic v.0.36, followed by alignment to the mm10 assembly with bowtie2 v.2.4.1. Low-quality (MAPQ < 30), mitochondrial genome and duplicated reads were removed using samtools v.1.9 and genome coverage calculation for visualization purposes on the UCSC genome browser was performed using deepTools v.3.5.1 excluding blacklisted regions. The bam files of WT and dKO samples were concatenated into a master bam file, used to call significant peaks with matching input controls using MACS2 v.2.1.0. Significance q-value cut-offs were determined post-hoc, testing several q-values based on signal to background ratio. Quantification of reads in significant peak for all samples was preformed using bedtools v.2.29.2 multicov. Traditional (TEs) and super-enhancers (SEs) were called based on H3K27ac enrichment using the ROSE v0.11 algorithm (Rank Ordering of Super-Enhancers) with stitching distance 12.5kb and TSS exclusion zone size 2.5kb. The ROSE algorithm was also used to extract H3K27ac levels at TEs and SEs for WT and dKO samples individually. Average H3K27ac signal across all elements was calculated for each sample and further used for normalization between samples. Log2 fold change ratio of normalized signal (-0.75 < log2FC < 0.75) was used to call differential TEs and SEs. All other enhancers were considered static.

Flow Cytometry

Plots

Confirm that:

☒ The axis labels state the marker and fluorochrome used (e.g. CD4-FITC).

☒ The axis scales are clearly visible. Include numbers along axes only for bottom left plot of group (a 'group' is an analysis of identical markers).

☒ All plots are contour plots with outliers or pseudocolor plots.

☒ A numerical value for number of cells or percentage (with statistics) is provided.

Methodology

Sample preparation

Resected BRAFV600E/PTEN-deficient melanomas were cut into 1-2mm fragments. For immunophenotyping and CD8+ T cell sorting, tumor fragments were digested in RPMI containing 400 U/ml collagenase IV (Gibco), 100 U/ml hyaluronidase (Sigma) and 100 μg/ml DNAse (Roche) at 37ºC for 1 hour, aspirated 5 times through a 14G needle, and filtered through a 70 μm cell strainer. Immune cells were enriched by centrifugation through a discontinuous 40/90 Percoll (GE Healthcare Life Sciences) gradient. For sCAF sorting, tumor fragments were digested using the Tumor Dissociation Kit, mouse (Miltenyi) in DMEM using the soft/medium protocol according to manufacturer's instructions.

Instrument

Analysis: LSRFortessa (BD Biosciences); sorting: FACSAria III SORP (BD Biosciences)

Software

FacsDiVa (BD Biosciences) v8.0.2

Cell population abundance

Sorting was performed in "purity" mode and sort performance was determined to be >95% using Accudrop beads.
Cell numbers were insufficient to determine purity for sorted CD8 T cells. Transcriptomic analysis performed on the sorted T cells suggests these are pure populations based on expression of key cell type-specific genes.
Similarly, all sorted CAFs were used for downstream studies. Sorted CAFs that went into culture were confirmed to be free of tumor cell contamination and expressed CAF marker PDGFRa (Supplemental Figure 4k, l).

Gating strategy

Single events were gated on FSC-A/FSC-W and SSC-A/SSC-W. Cells were gated on FSC-A/SSC-A. Live cells were gated on viability dye (DAPI for sorting, LIVE/DEAD Fixable Blue Dead Cell Stain for analysis) negativity. Immune cells were gated on CD45 positivity. Further gating is illustrated in Supplemental Figure 2c and follows the strategy used in Salmon et al., 2016 (https://pubmed.ncbi.nlm.nih.gov/27096321/). Boundaries between "positive" and "negative" staining cell populations were defined based on the histogram valley of the respective channel. When sorting CAFs from whole tumor single cell suspensions, immune cells present in each sample, expected to be positive for CD45 and negative for PDGFRa, were used to define PDGFRa gate boundaries.

☒ Tick this box to confirm that a figure exemplifying the gating strategy is provided in the Supplementary Information.
