# Supplementary material for: MacroH2A restricts inflammatory gene expression in melanoma cancer-associated fibroblasts by coordinating chromatin looping
Source: Nat Cell Biol. 2023 Aug 21;25(9):1332–45. doi: 10.1038/s41556-023-01208-7 (PMC10495263; doi:10.1038/s41556-023-01208-7)

Source Data for Figure 5f

CAF 5016
CAF 5680
CAF 6128
CAF 6977
CAF 7074
CAF 7138
CAF 10996
CAF 40
CAF 45
CAF 47
CAF 48
WT CAF (mouse)
dKO CAF (mouse)

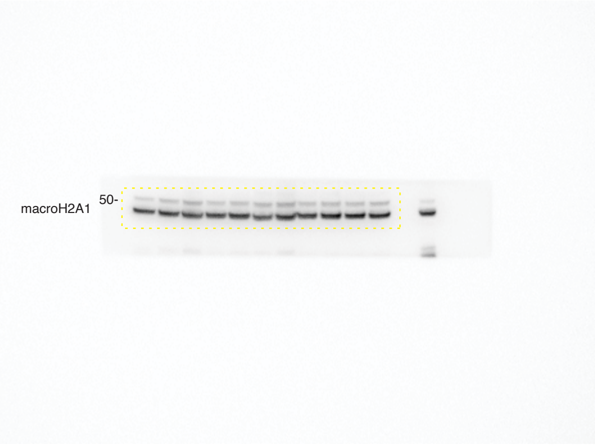

Note: total protein stain for this blot (below) not shown in main figure

CAF 5016
CAF 5680
CAF 6128
CAF 6977
CAF 7074
CAF 7138
CAF 10996
CAF 40
CAF 45
CAF 47
CAF 48
WT CAF (mouse)
dKO CAF (mouse)

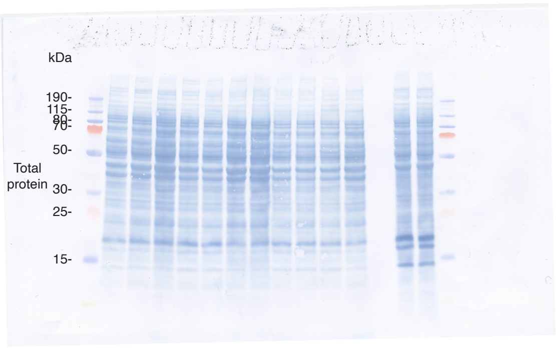

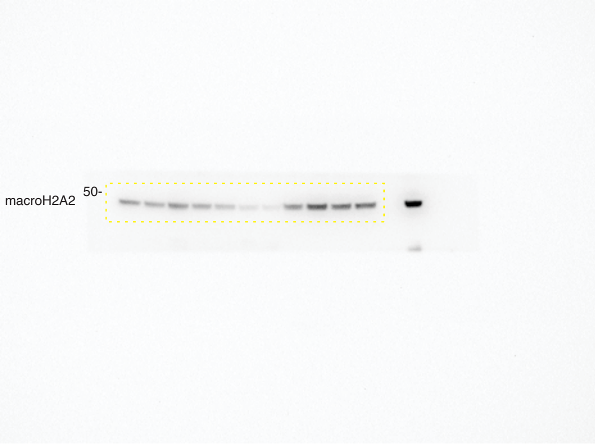

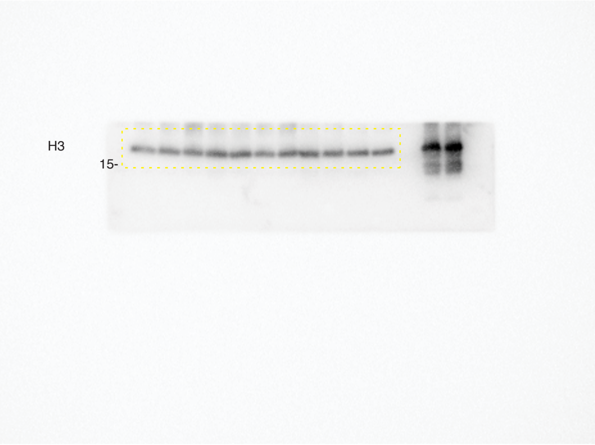

Supplement: Source Data Fig. 5 — Unprocessed western blots. [file 41556_2023_1208_MOESM16_ESM.pdf]
