# Supplementary figures and images for: MacroH2A restricts inflammatory gene expression in melanoma cancer-associated fibroblasts by coordinating chromatin looping
Source: Nat Cell Biol. 2023 Aug 21;25(9):1332–45. doi: 10.1038/s41556-023-01208-7 (PMC10495263; doi:10.1038/s41556-023-01208-7)

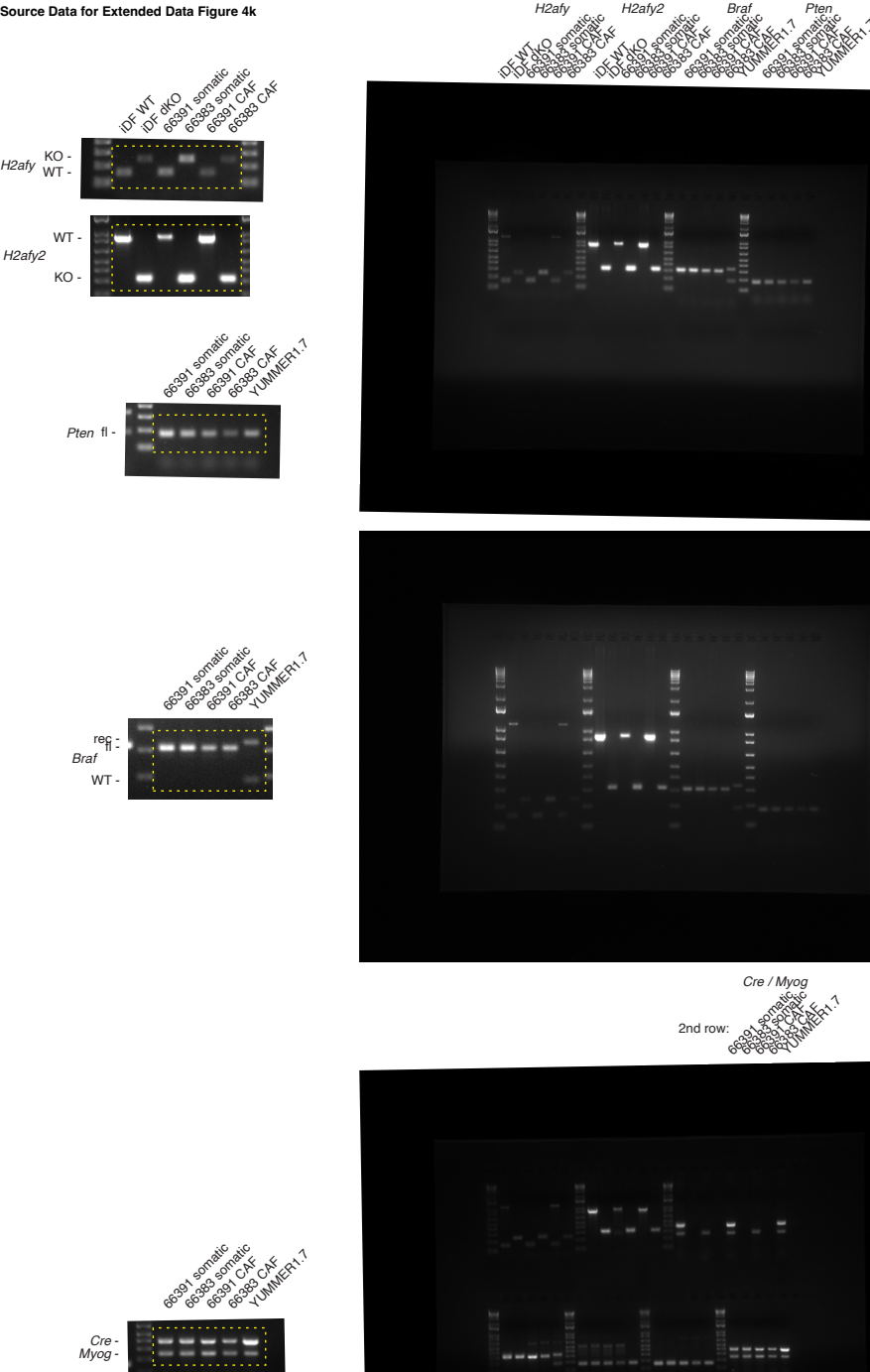

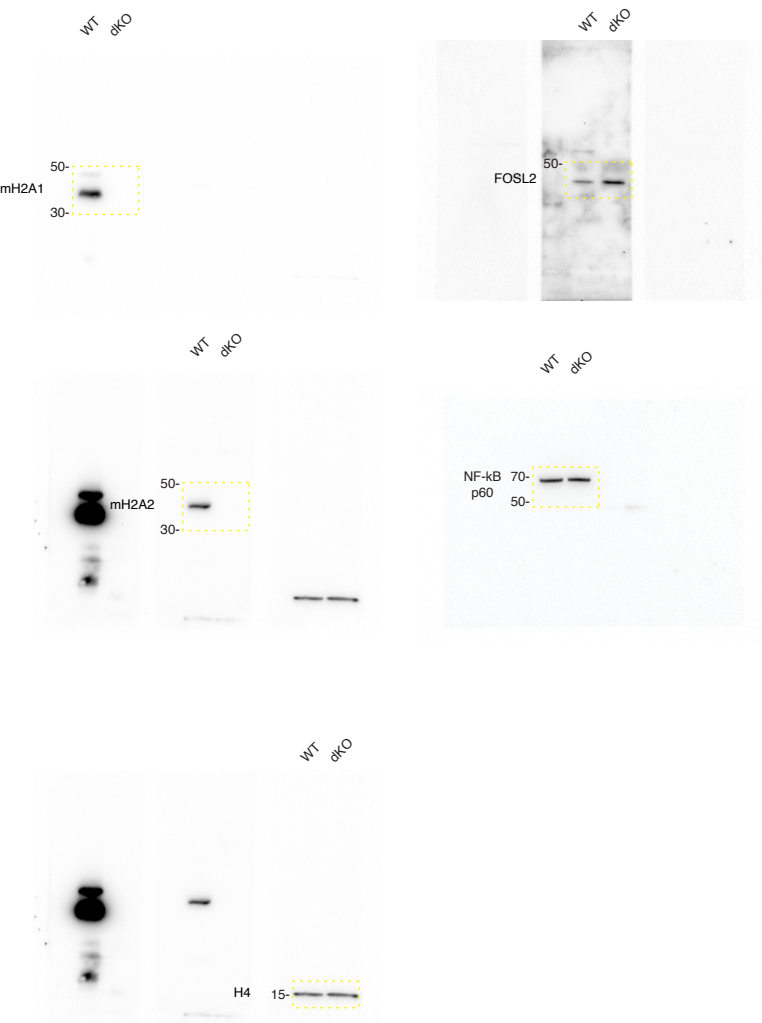

Supplement: Source Data Extended Data Fig. 4 — Unprocessed gels. [file 41556_2023_1208_MOESM23_ESM.pdf]

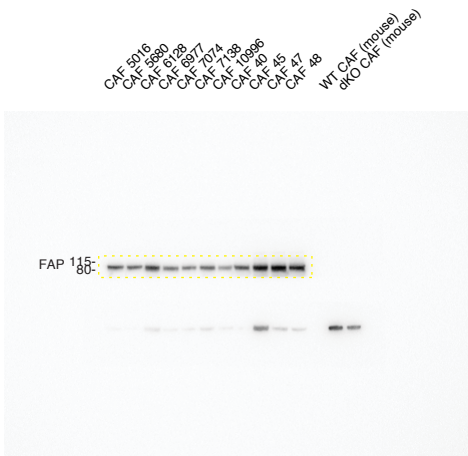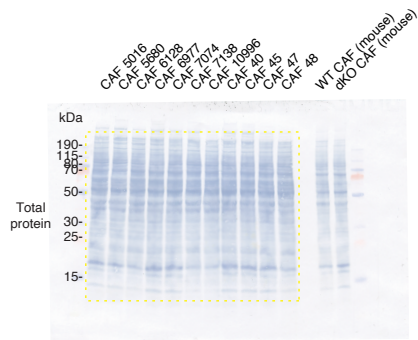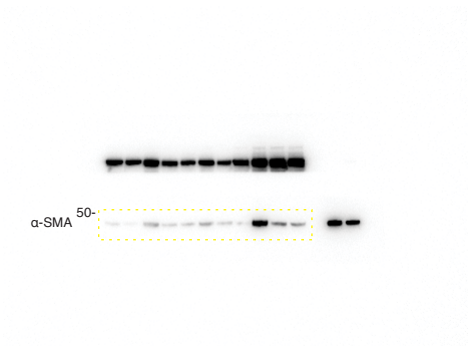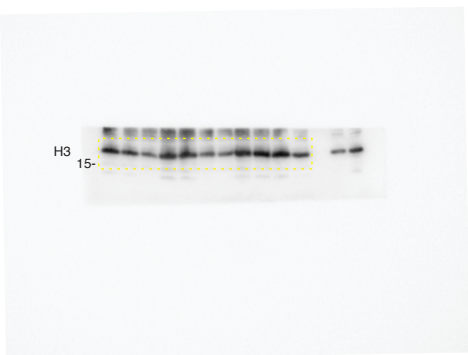

Supplement: Source Data Extended Data Fig. 5 — Unprocessed western blots. [file 41556_2023_1208_MOESM25_ESM.pdf]
